# Mice Deficient in CD38 Develop an Attenuated Form of Collagen Type II-Induced Arthritis

**DOI:** 10.1371/journal.pone.0033534

**Published:** 2012-03-16

**Authors:** Jorge Postigo, Marcos Iglesias, Daniela Cerezo-Wallis, Antonio Rosal-Vela, Sonia García-Rodríguez, Mercedes Zubiaur, Jaime Sancho, Ramón Merino, Jesús Merino

**Affiliations:** 1 Departmento de Biología Molecular, Instituto de Formación e Investigación Marqués de Valdecilla, Universidad de Cantabria, Santander, Spain; 2 Instituto de Biomedicina y Biotecnología de Cantabria/CSIC-Universidad de Cantabria-SODERCAN, Santander, Spain; 3 Instituto de Parasitología y Biomedicina López Neyra, CSIC, Granada, Spain; University of São Paulo, Brazil

## Abstract

CD38, a type II transmembrane glycoprotein expressed in many cells of the immune system, is involved in cell signaling, migration and differentiation. Studies in CD38 deficient mice (CD38 KO mice) indicate that this molecule controls inflammatory immune responses, although its involvement in these responses depends on the disease model analyzed. Here, we explored the role of CD38 in the control of autoimmune responses using chicken collagen type II (col II) immunized C57BL/6-CD38 KO mice as a model of collagen-induced arthritis (CIA). We demonstrate that CD38 KO mice develop an attenuated CIA that is accompanied by a limited joint induction of IL-1β and IL-6 expression, by the lack of induction of IFNγ expression in the joints and by a reduction in the percentages of invariant NKT (iNKT) cells in the spleen. Immunized CD38 KO mice produce high levels of circulating IgG1 and low of IgG2a anti-col II antibodies in association with reduced percentages of Th1 cells in the draining lymph nodes. Altogether, our results show that CD38 participates in the pathogenesis of CIA controlling the number of iNKT cells and promoting Th1 inflammatory responses.

## Introduction

The nicotinamide adenine dinucleotide (NAD^+^) glycohydrolase CD38 (EC 3.2.2.5) is a type II transmembrane glycoprotein widely expressed in many cell population of the immune system, including B and T cells, NK cells, circulating monocytes and DC as well as in non-hematopoietic cells [1], [2]. This molecule acts as an ectoenzyme that catalyzes the formation of adenosine diphosphate ribose (ADPR), cyclic ADPR (cADPR), and nicotinamide from NAD^+^ under neutral pH; or nicotinic acid adenine dinucleotide phosphate (NAADP^+^) from NADP^+^ under acidic conditions [1]–[5]. Both cADPR and NAADP^+^ are potent endogenous activators of intracellular Ca^2+^ release and function as signaling molecules in leukocytes and other CD38 expressing non-hematopoietic cells [6]. In addition to its ectoenzyme activity, CD38 can also function as a plasma membrane signaling receptor in leukocytes [2], [7] interacting with CD31/PECAM-1 expressed by endothelial cells and other cell lineages. This interaction promotes leukocyte proliferation, T cell activation, monocyte-derived DC maturation, survival and migration and induces Th1 polarization in co-cultures of DC with CD4^+^ T lymphocytes [8]–[10]. In this regard, our studies indicate that CD38 is located in privileged sites for signaling and cell-communication such as membrane rafts, immunological synapse, recycling endosomes, and exosomes [10]–[13]. Moreover, CD38 signaling potential varies depending upon the cellular context and its physical and/or functional association with other signaling molecules [10], [12], [13].

Studies in CD38 deficient mice (CD38 KO mice) highlight the importance of this molecule for the appropriated functioning of the immune system. CD38 deficiency has been associated with defects in humoral B-cell responses [14], [15], neutrophil migration [16] and DC trafficking [15]. In CD38 KO mice, the numbers of peripheral Tregs and invariant NKT (iNKT) cells are reduced as a result of a NAD^+^-induced cell death process [17], [18]. The extracellular accumulation of NAD^+^ occurring in these mice induces the ADP ribosyltransferase-2 (ART-2)-mediated ADP-ribosylation of the P2X7 purinergic receptor and its ATP-independent activation which initiates the apoptotic process [19]. Thus, CD38 acts as a critical regulator of inflammatory and innate immune responses and CD38 deficiency in NOD mice accelerates the development of type I diabetes (T1D) [17]. In NOD mice activation of iNKT cells with the superagonist alpha-galactosylceramide leads to differentiation of tolerogenic DC, which inhibits the development of T1D [18]. In contrast, in the absence of CD38, ART-2 preferentially activates apoptotic deletion of CD4^+^ iNKT cells and accelerates T1D onset [18]. However, it should be stressed that iNKT cells through the production of IL-17 may also have pro-inflammatory effects as occurs during the development of collagen type II-induced arthritis (CIA) where mice deficient or depleted in such cells develop an attenuated form of disease [20], [21]. Moreover, activation of iNKT cells in the C57BL/6 (B6) background, unlike in the NOD genetic background, has an adjuvant-like effect that enhances various immunological responses including the downstream differentiation of non-tolerogenic DCs [22]. In this regard, CD38 KO mice in the B6 genetic background develop milder inflammatory lesions in a model of post-ischemic inflammation and brain injury after temporary middle cerebral artery occlusion, although a direct relationship between this protective effect and changes in iNKT cells has not been established [23]. Inflammatory responses and airway hyperreactivity are also attenuated in allergen-challenged CD38 KO mice [24], [25]. Moreover, in SLE patients increased numbers of CD38^+^ B cells have been observed and in patients with active disease, B cells expressing high levels of CD38 produce IgG anti-dsDNA autoantibodies [26].

Based in these apparently conflicting results, in the present study we have explored the contribution of CD38 to the control of autoimmunity using the experimental model of collagen type II (col II)-induced arthritis (CIA) in CD38 KO mice. We demonstrate here that in comparison to WT mice, CD38 KO mice develop an attenuated form of CIA in association with lower percentages of iNKT cells and a down-modulation in Th1 immune responses.

## Results

### Development of an attenuated CIA in CD38 KO mice

**Figure 1:**
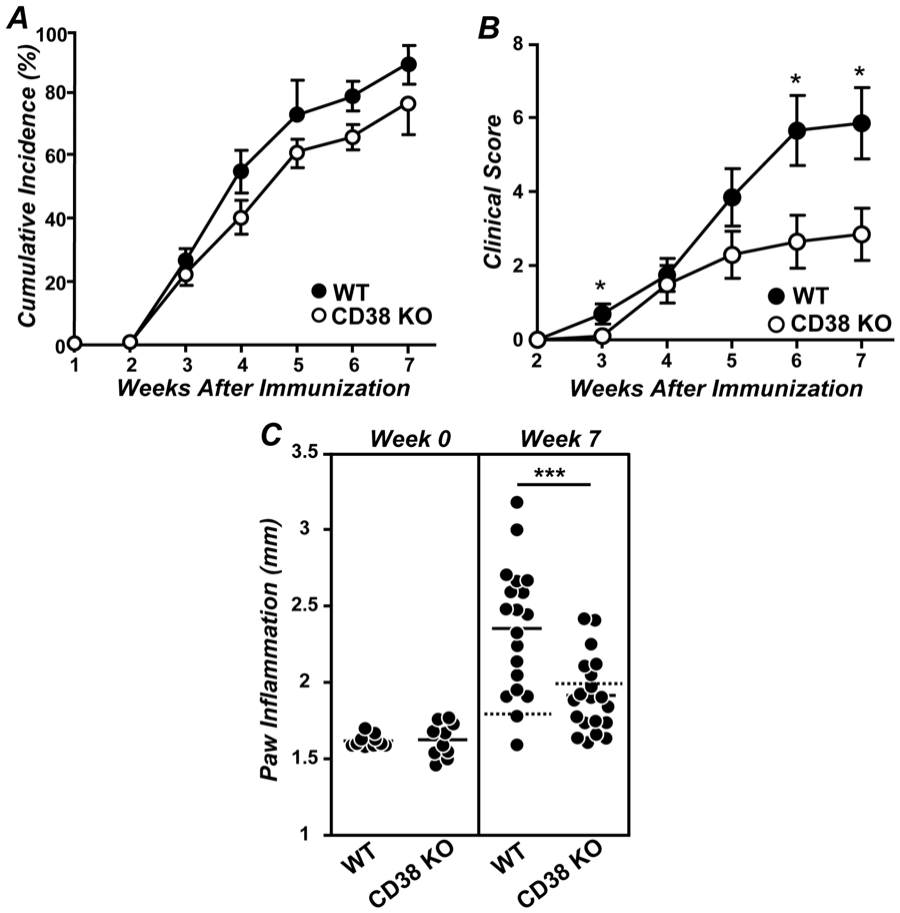
Involvement of CD38 in the development of CIA. (A) Cumulative incidence of CIA. Results are expressed as the mean ± SD of the percentage of affected mice at the indicated weeks after immunization. (B) Clinical severity of CIA at different periods after immunization with col II-CFA. Results are expressed as the mean ± SD of the clinical score in each mouse. (C) Paw inflammation was measured 7 weeks after immunization using a digital caliper. The mean values of the inflammation in the four paws of every individual mouse are expressed. Solid bars represent the mean value of each examination. Dotted bars represent the mean ± 3SD of the values found in their respective non-immunized control groups. Statistic differences between WT and CD38 KO mice are indicated as follow: *p<0.05, **p<0.01, *** p<0.001.

**Figure 2:**
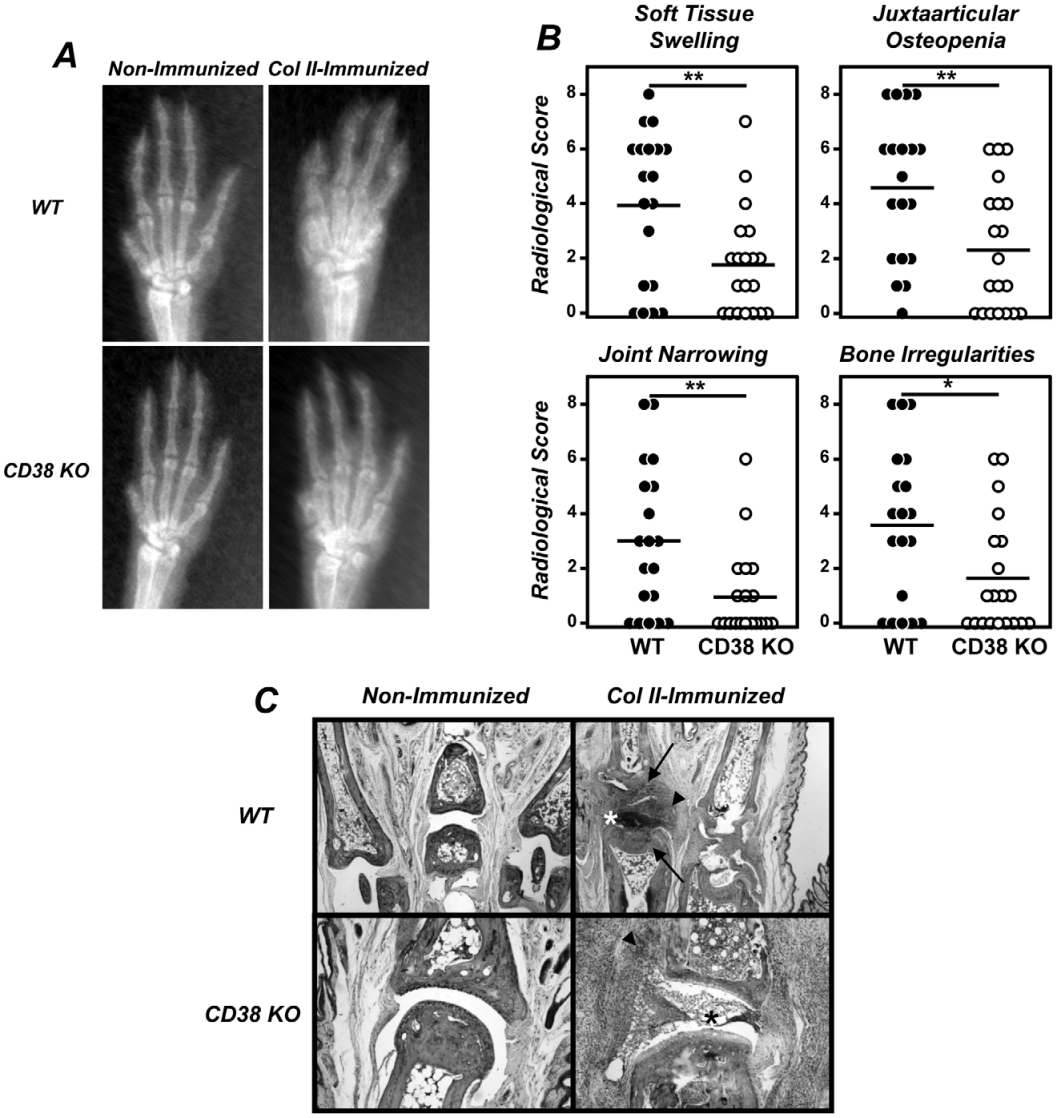
Reduced radiological and histological lesions in CIA developing CD38 KO mice. 8–12 weeks-old WT and CD38 KO male mice were immunized with col II-CFA. (A) Representative radiological images of the front paws of WT and CD38 KO mice, non-immunized and 7 weeks after immunization with col II-CFA. (B) Radiological score of four individual signs. Results are expressed as the values of individual mice. Bars represent the mean value of each examination. Statistic differences are indicated as follow: *p<0.05, **p<0.01. (C) Representative histological appearance of the joints (×10) of WT and CD38 KO mice non-immunized and 7 weeks after immunization with col II-CFA. Signs of cartilage and/or bone destruction (arrows), sinovitis (arrow head) and pannus (*) are indicated.

In the present study we explored whether the deficiency in CD38 influenced the clinical progression of CIA in B6 mice. To this end, we immunized WT and CD38 KO mice with chicken col II-CFA. The cumulative incidence of CIA was slightly, although not significantly, lowers in CD38 KO mice than in WT mice (Figure 1A). However, the clinical severity of CIA was also lower in CD38 KO mice than in WT mice (Figure 1B). To further analyze the degree of paw inflammation during CIA development, the thickness of the paws at the level of the metacarpus and metatarsus was measured with a digital caliper 7 weeks after col II-immunization. A 90% of WT mice (19/21 mice) exhibited a significant inflammation of the paws (inflammation values higher than the mean ± 3SD of those found in non-immunized controls), whereas only 30% (6/20 mice) of col II-immunized CD38 KO mice showed signs of paw inflammation (Figure 1C). In correlation with the clinical findings, the severity of every individual radiological sign considered here (soft tissue swelling, juxtaarticular osteopenia, narrowing or disappearance of the interosseous spaces reflecting cartilage loss and bone irregularities secondary to periosteal new bone formation and/or marginal articular erosions) was significantly lower in CD38 KO than in WT mice (Figure 2A and B). The histological analyses of the joints in CD38 KO mice failed to show signs of cartilage destruction although showed the presence of synovitis and pannus formation (Figure 2C). All these lesions were present in WT mice (Figure 2C).

### Reduced number of iNKTs and lack of involvement of Tregs in the protection against CIA of CD38 KO mice

**Figure 3:**
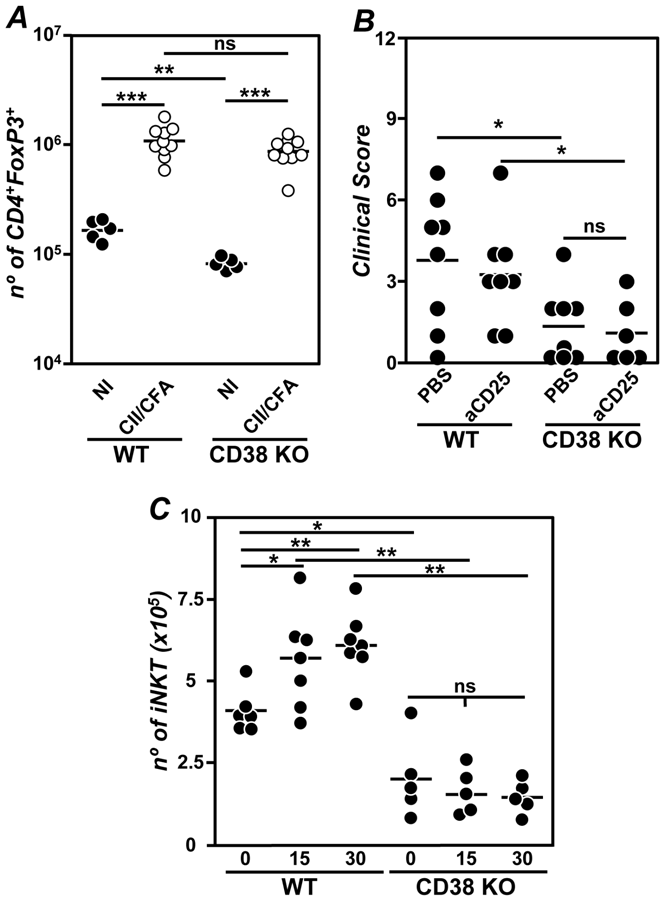
Lack of involvement of Tregs in the reduced CIA of CD38 KO mice. (A) Number of Tregs in the lymph nodes of WT and CD38 KO mice non-immunized (NI; •) and 3 weeks after immunization with col-II-CFA (○). Representative results of 3 independent experiments are expressed as the values of individual mice. Bars represent the mean value of each examination. (B) Clinical severity of CIA 7 weeks after immunization with col II-CFA in PBS-treated or Treg-depleted WT and CD38 KO mice Representative results of 2 independent experiments are expressed as the values of individual mice are expressed. Bars represent the mean value of each examination. C) Number of iNKT cells in the spleen of WT mice and CD38 KO mice before (day 0) and 15 and 30 days after col II immunization. Results are expressed as mean ± SD. Statistic differences are indicated as follow: ns: non-significant, *p<0.05, **p<0.01, ***p<0.001.

It has been reported that Tregs are very susceptible to the pro-apoptotic effects of extracellular NAD^+^ and accordingly, CD38 KO mice exhibit low numbers of this cell population [17]. In agreement with these results, the number of lymph node Tregs in our non-immunized CD38 KO mice were significantly lower than in WT mice (Figure 3A). However, this cell population increased to similar levels in both CD38 KO and WT mice after col II immunization (Figure 3A).

We then explored whether the increase in the number of Tregs after the immunization with col II-CFA in CD38 KO mice was involved in the protection against CIA. To this end, groups of WT and CD38 KO mice were either depleted in Tregs with a citotoxic anti-CD25 mAb or treated with PBS as a control, prior to the induction of CIA. Again, the severity of CIA was lower in PBS-treated CD38 KO than in PBS-treated WT mice and this severity did not increase after depletion of Tregs in both groups of mice (Figure 3B).

Similarly to Tregs, iNKT cells are also very susceptible to the pro-apoptotic effects of extracellular NAD^+^ [18]. In this regard, the number of iNKT cells in the spleen of non-immunized CD38 KO mice was severely reduced in comparison to WT mice (Figure 3C). However, unlike Tregs, the immunization with col II-CFA induced a significant increase of iNKT cells in WT, but not in CD38 KO mice (Figure 3C).

### Altered anti-col II antibody production in CD38 KO mice

**Figure 4:**
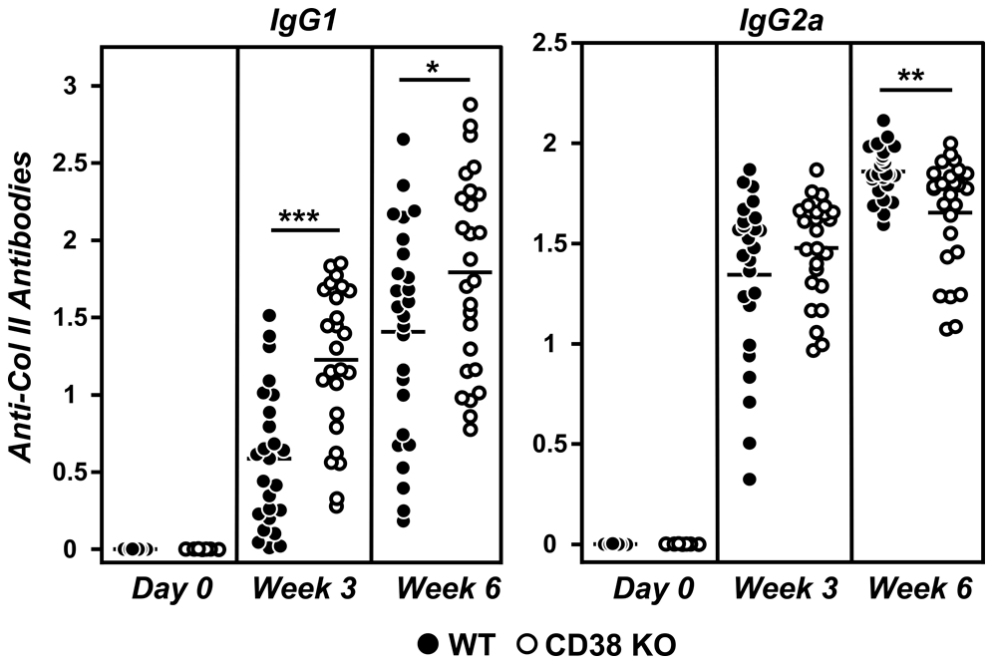
Serum levels of IgG1 and IgG2a anti-col II antibodies before, 3 and 8 weeks after immunization with col II-CFA. Values of individual mice are expressed. Bars represent the mean value of each examination. Statistic differences are indicated as follow: *p<0.05, **p<0.01, ***p<0.001.

The intensity and quality of anti-col II humoral immune responses was compared between col II-immunized CD38 KO and WT mice by analyzing the levels of circulating IgG1 and IgG2a anti-col II antibodies. Both groups of mice exhibited strong anti-col II antibody responses 3 and 6 weeks after CIA-induction (Figure 4). However, qualitative differences in these antibody responses were observed between CD38 KO and WT mice. Thus, serum levels of IgG1 anti-col II antibodies were significantly higher at both 3 and 6 weeks after immunization in CD38 KO mice as compared with those in WT mice. In contrast, the levels of IgG2a anti-col II antibodies were significantly higher in WT than in CD38 KO mice at 6 weeks upon immunization, whereas the differences were not statistically significant at the onset of the disease (Figure 4), suggesting that CD38 KO mice show an altered Th1/Th2 balance in response to col II immunization.

### Reduced joint expression of pro-inflammatory cytokines and altered Th1 cytokine pattern in CD38 KO mice

**Figure 5:**
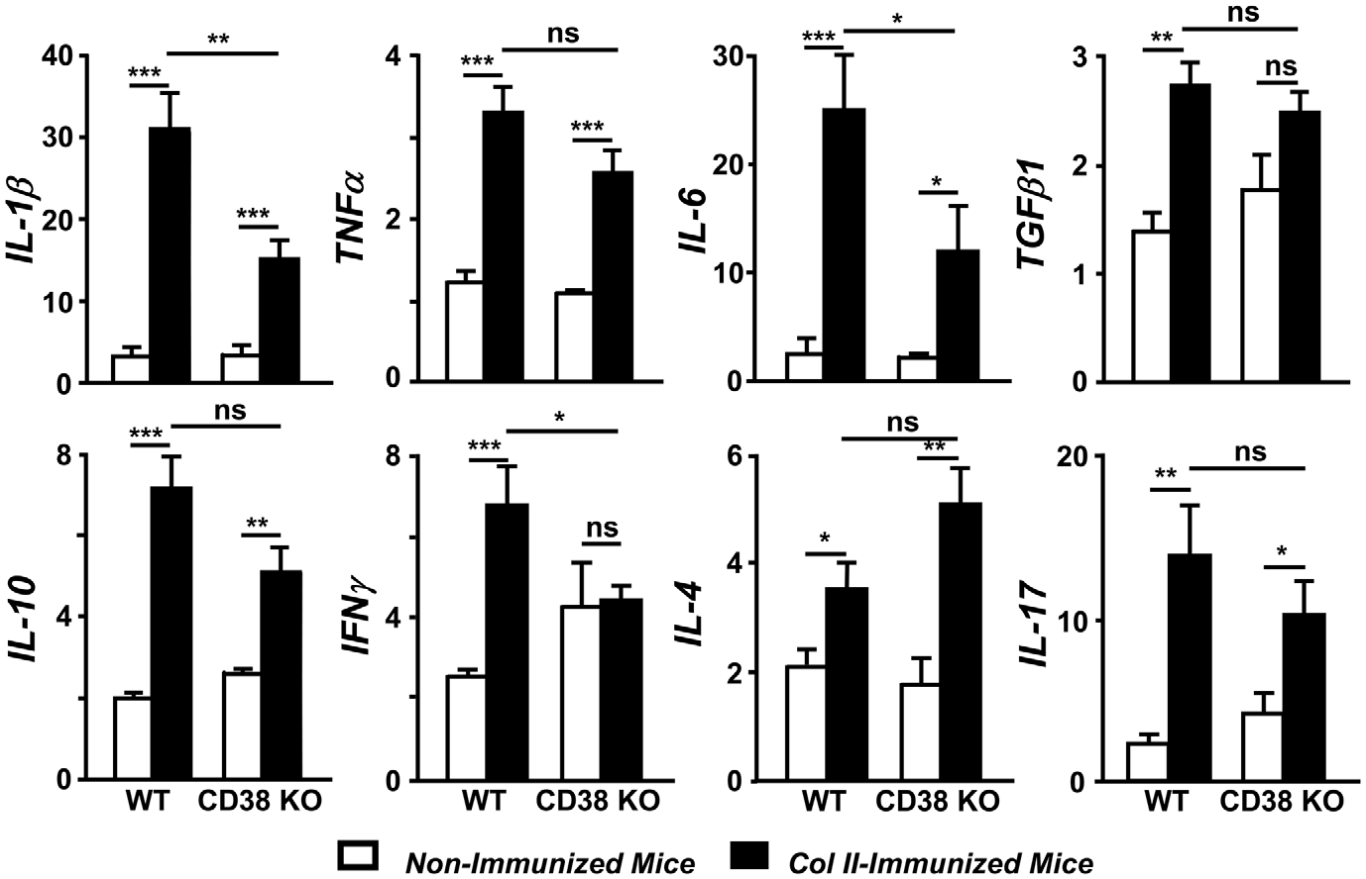
Cytokine expression pattern in the joints of WT and CD38 KO mice during CIA development. Analysis by quantitative real-time RT-PCR of mRNA from different cytokines in the paws of WT and CD38 KO mice, non-immunized (open bars) and 7 weeks after immunization with col II-CFA (closed bars). Results from three independent experiments (15–20 animals/group) are expressed as mean ± SD fold change of each cytokine relative to GAPDH expression measured in parallel in each sample. Statistic differences are indicated as follow: ns: non-significant, *p<0.05, **p<0.01, *** p<0.001.

The reduced severity of CIA in CD38 KO mice together with the altered anti-col II antibody responses compared to WT mice, prompted us to explore by quantitative real time RT-PCR the pattern of cytokine expression in the joints during the induction of CIA in these strains of mice. As expected [27], a significant increase in the expression of the arthritogenic IL-1β, TNFα and IL-6 transcripts was observed in the joints of WT mice 7 weeks after immunization with col II-CFA (Figure 5). In addition, joint levels of TGFβ1, IL-10, IFNγ, IL-4 and IL-17 mRNAs were augmented in WT mice with arthritis (Figure 5). In parallel with the clinical, radiological and histological findings, the expression of IL-1β and IL-6, but not of TNFα was reduced in the joints of col II-immunized CD38 KO mice (Figure 5). The reduced severity of CIA in CD38 KO mice was not accompanied by an increased joint expression of the TGFβ1 and IL-10 inhibitory cytokines (Figure 5). Unlike WT mice, the expression of IFNγ, the prototypical Th1 cytokine, was not induced in the joints of CD38 KO mice after CIA induction (Figure 5) Although the differences were not statistically significant, the induction in the expression of IL-4 and IL-17 and in the joints of CD38 KO mice was higher and lower, respectively, than the observed in WT mice (Figure 5).

### Differential induction of Th1, but not Th17 cells after CIA induction in CD38 KO and WT mice

**Figure 6:**
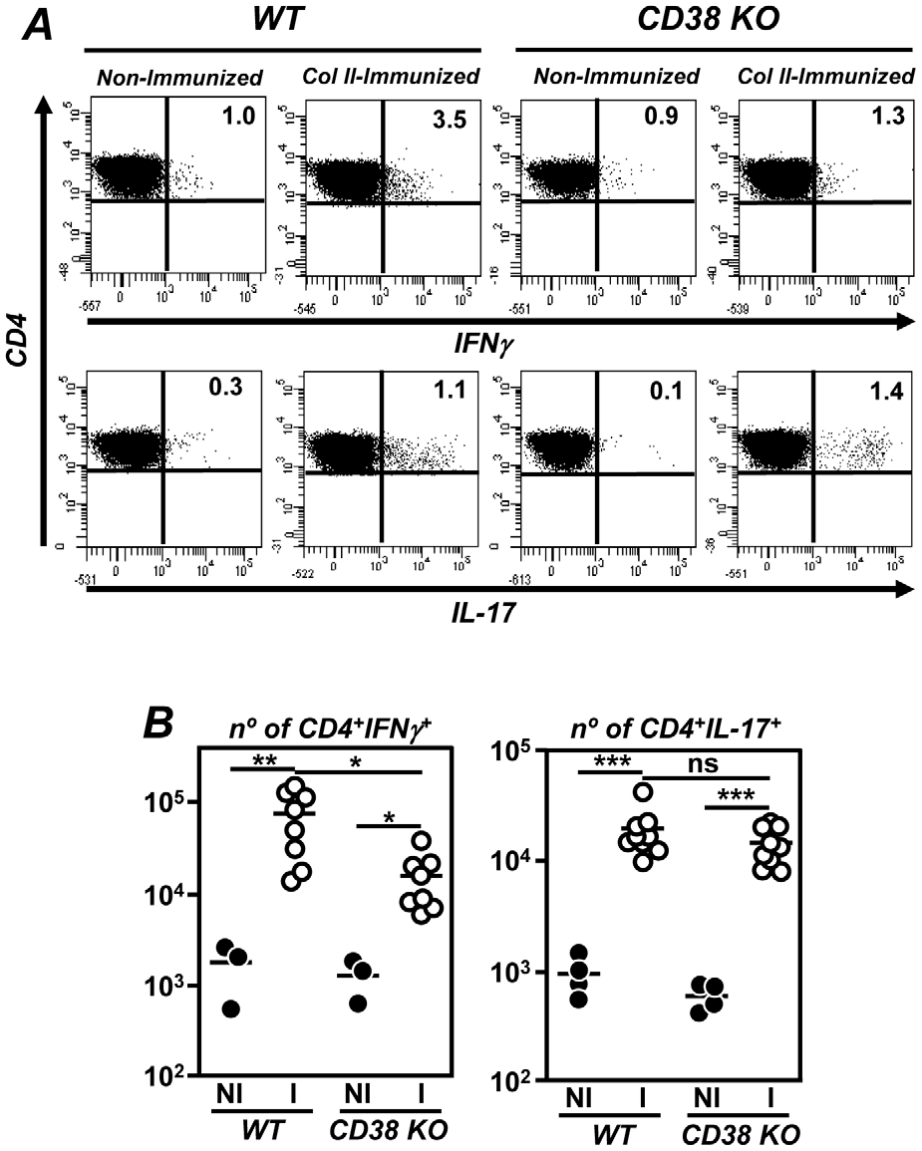
Expansion of Th17 but not and Th1 cells in the draining lymph nodes of col II-immunized CD38 KO mice. WT and CD38 KO mice male mice were immunized with col II-CFA. For intracellular cytokine staining, lymphocytes from paraaortic lymph nodes were stimulated 3 weeks after immunization with phorbol myristate acetate and ionomycin in the presence of GolgiStop solution. (A) Representative histograms of CD4^+^IFNγ^+^ Th1 and CD4^+^IL-17^+^ Th17 cells, determined by flow cytometry, in the different experimental groups. The percentage of each population is indicated. (B) Number of CD4^+^IFNγ^+^ Th1 (left panel) and CD4^+^IL-17^+^ Th17 (right panel) cells in the draining lymph nodes of non-immunized (NI; •) and 3 weeks post-immunized (○) mice. Values of individual mice are expressed. Bars represent the mean value of each examination. Statistic differences are indicated as follow: ns: non-significant, *p<0.05, **p<0.01, *** p<0.001.

Due to the pattern of cytokine expression in the joints and to further explore the mechanisms implicated in the amelioration of CIA in CD38 KO mice, we compared the number of different functional CD4^+^ T-cell subpopulations in the draining lymph nodes of WT and CD38 KO mice before and after immunization with col II-CFA. Both IFNγ-producing Th1 and IL-17-producing Th17 cell populations were augmented in the paraaortic lymph nodes of WT mice 3 weeks after immunization with col II-CFA (Figure 6). A similar increase in the number of Th17 cells was observed in CD38 KO mice. Although the number of CD4^+^IFNγ Th1 cells in the draining lymph nodes of immunized CD38 KO mice was also higher than in non-immunized mice, such an increase was significantly lower than the observed in immunized WT mice in correlation with the reduced IFNγ expression in the joints and of circulating IgG2a anti-col II antibody responses (Figure 6).

## Discussion

Different studies in experimental murine models of inflammatory/autoimmune diseases reveal some discrepancies about the role of CD38 in the control of pathological immune responses [17], [18], [23]–[26]. Here, we extend these previous observations by analyzing the contribution of CD38 to the pathogenesis of CIA, a paradigmatic model of autoimmune disease mediated by Th1 and Th17 lymphocytes. Our results show that the severity of CIA in CD38 KO mice is lower than in WT mice. These results clearly contrast with the development of accelerated diabetes in NOD mice deficient in CD38, partly because of a loss of iNKT cells and Tregs [17], [18] that are highly sensitive to NAD-induced cell death activated by ART-2-mediated ADP ribosylation of P2X7 receptors [19]. However, after immunization of CD38 KO mice with col II-CFA, the number of Tregs reaches that of immunized WT mice. Although the reasons for the increase in Treg observed in immunized CD38 KO mice are not clear yet, the experiments in animals depleted in these cells after treatment with a cytotoxic anti-CD25 mAb clearly indicate that this regulatory T cell population does not play a role in the protection against CIA observed in such mice. One intriguing finding in our study is the lack of exacerbation of CIA in Treg-depleted WT mice which contrasts with the increase in arthritis severity after anti-CD25 treatment observed by others [28], [29]. However, these discrepancies can be attributed to the different models of inflammatory arthritis used in these studies and/or to the different timing of anti-CD25 mAb administration during the development of the disease. In this regard, in our study the anti-CD25 treatment is administered once prior to the induction of CIA instead of the 4 weeks-long treatment initiated 13 days after col II-immunization used by Kelchtermans et al [28]. In addition, the treatment protocol used here has been proved to be able to reverse the protection against CIA in transgenic mice overexpressing human Bcl-2 in T cells [30], further indicating that the lack of exacerbation of CIA in Treg-depleted WT mice is not related to the concomitant possible depletion of pathogenic CD4 cells expressing transitory CD25 after their activation.

Similarly to Tregs, the number of iNKT cells, that also possess regulatory properties in certain models of autoimmune diseases, is also reduced in CD38 KO mice [18]. This reduction has been involved in the acceleration and aggravation of T1D in NOD mice [17]. However, iNKT cells through the production of IL-17 promote CIA development and mice deficient or depleted in such cells develop an attenuated form of disease resembling that of our B6 CD38 KO mice [20], [21]. Moreover, activation of iNKT cells in the B6 background, unlike in the NOD genetic background, has an adjuvant-like effect that enhances various immunological responses including the downstream differentiation of non-tolerogenic DCs [22]. It is, therefore, likely that the reduction in the number of iNKT cells observed in CD38 KO mice, that are not recovered during CIA development, eventually contribute to the development of a mild arthritis in CD38 KO. According to this possibility, the consequences of CD38 deficiency in the acceleration or attenuation of different inflammatory processes may be in part related to the respective anti- or pro-inflamatory role of iNKT cells in such diseases.

The development of an attenuated CIA in CD38 KO mice can be also related with the capacity of CD38 to regulate the migration of DC precursors from the blood to peripheral sites as well as the migration of mature DCs to lymph nodes in response to CCL2, CCL19, CCL21, and CXCL12 chemokines [15] affecting in that way the correct priming of CD4^+^ T cells. In addition, considering that cADPR and NAADP^+^ are potent endogenous activators of intracellular Ca^2+^ release [6], the deficiency of CD38 may impairs the activation of T cells. In this regard, CD38 KO mice immunized with low doses of T-dependent antigens precipitated with alum, mount poor IgG1 antigen specific humoral immune responses [15]. However, the development in col II-immunized CD38 KO mice of strong collagen-specific humoral immune responses and the induction of a similar Th17 response than the observed in WT mice argues against the possibility of poor T-cell priming as the cause of the attenuated CIA in these CD38 deficient mice.

Studies performed in animals immunologically depleted or genetically deficient in B cells underlined the importance of humoral immune responses in the induction of CIA [31], [32]. Strikingly, CD38 KO mice developing a less severe CIA than WT mice, exhibited higher serum levels of IgG1 and lower levels of IgG2a, anti-col II antibodies. The increase in the levels of IgG1 anti-col II antibodies observed in immunized CD38 KO mice contrasted with a previous report showing an impaired IgG1 humoral immune responses against T-dependent antigens in these mutant mice [15]. This discrepancy was probably attributed to the different immunization regimen used in both studies; the protocol used here for the induction of CIA required the use of high doses of col II emulsified in a *mycobacterium tuberculosis* enriched CFA instead of the immunization with very low doses of antigen precipitated with alum used in the former study. In fact, a direct comparison of humoral immune responses in CD38 KO mice reveals that the intensity of IgG1 immune responses, but not of IgG2a, IgG2b, IgG3 and IgA humoral responses, to T-dependent antigens was highly dependent on both the dose of the antigen and the adjuvant employed in the immunization protocol [14]. Studies addressing the pathogenic activity of autoantibodies revealed the importance of the IgG subclass in their capacity to promote tissue injury. Thus, it was demonstrated that the IgG2a switch variant of an anti-red blood cell autoantibody had about 20 times more pathogenic potential than the IgG1 switch variant [33], in clear correlation with the different affinities of IgG2a and IgG1 antibodies for Fcγ receptors promoting antibody-dependent cellular cytotoxicity [34] and with the higher capacity of IgG2a antibodies to activate the complement cascade [35]. In addition to the possible differential ability to promote tissue injury, the changes in the levels of circulating IgG1 and IgG2a anti-col II antibodies observed in CD38 KO mice compared to WT mice indirectly reflected a distinct pattern of functional CD4^+^ T cell differentiation between both strains of mice after col II immunization.

Whereas the pathogenic role of Th17 cells in the development of CIA has been clearly defined [36], [37], the contribution of Th1 cells is less clear. Thus, IFNã deficiency renders the normally resistant B6 strain susceptible to disease [38], [39], and lack of IFNã or signalling through the IFNã receptor enhances the severity of arthritis in susceptible strains such as DBA/1 [40], [41]. On the other hand, enhanced Th1 and Th17 immune responses are observed in experimental situations associated to exacerbated CIA, such as in mice with a deficiency of myeloid cell-specific IL-1 receptor antagonist or mice deficient in ApoE lipoprotein [42], [43]. In agreement with these last observations, joint expression of IFNã in col II immunized CD38 KO mice is similar to that of non-immunized controls and the induction of Th1 lymphocytes in the draining lymph nodes of these mice is significantly lower than in col II immunized WT mice. The contribution of CD38 to Th1 differentiation has been demonstrated previously. Disruption of CD38/CD31 interaction by soluble CD38 in LPS-stimulated monocyte-derived DC, co-cultured with naïve T cells, inhibits the release of IFNã by T cell and impairs Th1 polarization [8]. In addition, the absence of CD38 renders mice more susceptible to *mycobacterial avium* infection secondary to an ineffective Th1 differentiation and polarization, which is essential for the control of *mycobacterial avium* infection [9]. An additional mechanism accounting for the reduced CIA severity in CD38 KO mice is most likely related to the deficient induction of several arthritogenic cytokines in the joints such as IL-1β and IL-6 although the joint expression of TNFα is similar in both groups of CIA-developing mice.

All together, the data presented here underline the importance of CD38 in the pathogenesis of CIA probably by increasing the percentages of iNKT cells and by promoting the development of Th1 inflammatory responses. These results can be useful for the design of new therapeutic strategies for rheumatoid arthritis in humans.

## Materials and Methods

### Ethics Statement

All studies with live animals were approved by the Universidad de Cantabria Institutional Laboratory Animal Care and Use Committee in compliance with the Guide for the Care and Use of Laboratory Animals (ILAR, 1985).

### Mice

B6 WT mice were purchased from Harlan Iberica (Barcelona, Spain). CD30 KO mice were backcrossed onto the B6 background for more than 12 generations, as described previously [14]. Mice were bled from the retro-orbital plexus 3 and 6 weeks after immunization.

### Induction and assessment of arthritis and serological studies

Chicken col II (Sigma, St Louis, MO) was dissolved at a concentration of 2 mg/ml in 0.05 M acetic acid and emulsified with CFA containing 4 mg/ml of *Mycobacterium tuberculosis* (MD Bioproducts, Zürich, Switzerland). For the induction of CIA, 8–12 weeks-old male mice were immunized once at the base of the tail with 150 µg of antigen in a final volume of 150 µl. The *in vivo* depletion of Tregs was performed using an anti-CD25 mAb (PC61: rat IgG1). Mice received a single injection of 2.5 mg of anti-CD25 mAb or PBS as controls, 4 days prior the immunization with col II. The efficiency of this treatment was evaluated in peripheral blood mononuclear cells by flow cytometry the one day before immunization. Previous studies showed that this treatment was able to reverse the protection against CIA in transgenic mice overexpressing human Bcl-2 in T cells [30]. An evaluation of arthritis severity was performed weekly. Clinical severity was quantified according to a graded scale of 0–3 as follows: 0 = no inflammation (normal joint); 1 = detectable local swelling and/or erythema; 2 = swelling in >1 joint and pronounced inflammation; 3 = swelling of the entire paw and/or ankylosis. Each paw was graded, and the scores were summed (with a maximum possible score 12 per mouse). In addition, the inflammation of the paws was measured at the level of metacarpus and metatarsus 7 weeks after immunization using a digital caliper (Somet CZ, Bilina, Czech Republic).

Radiological studies were performed using a CCX Rx ray source of 70 Kw with an exposition of 90 ms (Trophy Irix X-Ray System; Kodak Spain, Madrid) and Trophy RVG Digital Imagining system as previously described [42]. Radiological images were scale graded according to the presence of 4 different radiological lesions (1: soft tissue swelling, 2: juxtaarticular osteopenia due to alterations in bone density, 3: joint space narrowing or disappearance and 4: bone surface irregularities due to marginal erosions and/or periosteal new bone formation). The extension of every individual lesion in each paw (local: affecting one digit or one joint in the carpus; diffuse: affecting two or more digits and/or two or more joints in the carpus) was graded from 0 to 2 as follow: 0: absence; 1: local; 2: diffuse.

Mice were killed 7 weeks after immunization and the hind paws were fixed in 10% phosphate-buffered formaldehyde solution, decalcified in Parengy's decalcification solution overnight. The tissue was then embedded in paraffin. Sections (5 µm) were stained with hematoxylin and eosin and examined in a light-phase microscope.

Serum levels of IgG1 and IgG2a anti-col II antibodies were measured by ELISA as described [44]. The assays were developed with anti-mouse IgG subclass specific antibodies conjugated to alkaline phosphatase (Sigma). Results were expressed in Absorbance Units at 405 nm.

### Flow cytometry studies

According to the route of immunization (the base of the tail), the paraaortic lymph nodes were used as draining lymph nodes [45]. As expected, the size of these draining lymph nodes was greatly enlarged in both groups of mice after immunization with col II-CFA (number of paraaortic lymph node cells in WT mice before: 0.9±0.2×10^6^, and after immunization with col II-CFA: 5.9±0.3×10^6^; number of paraaortic lymph node cells in CD38 KO mice before: 1.2±0.1×10^6^, and after immunization with col II-CFA: 6.3±0.4×10^6^). The number of Tregs, Th1 and Th17 cells in the draining lymph nodes of WT and CD38 KO mice before and 3 or 7 weeks after immunization with col II-CFA were determined by flow cytometry, as described previously [42]. Briefly, intracellular cytokine staining was performed using an intracellular staining kit (BD Biosciences). Lymphocytes were stimulated with PMA (50 ng/ml) and ionomycin (750 ng/ml) in the presence of GolgiStop solution (BD Biosciences) for 6 hours and stained with FITC–conjugated anti-CD4 and PE-conjugated anti-IFNã or PE-conjugated anti–IL-17 (all antibodies from BD Biosciences). A PE-conjugated IgG2a irrelevant antibody was used as an isotype control of cytokine staining. Since in our non-immunized mice iNKT cells were almost undetectable in the paraaortic lymph nodes, the evaluation of iNKT cells was performed in the spleen at different time points after col II immunization. Cells were stained with PE-conjugated CD1d tetramers loaded with α-galactosylceramide (ProImmune, Oxford Science Park, UK) according to the manufacturer guidelines, FITC–conjugated anti-CD3 and allophycocyanin-conjugated CD19. The CD1d restricted iNKT cell population was identified as CD19^−^CD3^+^CD1d-tetramer-α-galactosylceramide^+^ cells. A total of 5×10^4^ viable cells were analyzed in a FACSCanto II flow cytometer using FACSDiva software (BD Biosciences).

### Quantitative real-time RT-PCR analyses

Total RNA was obtained from joints by TRIzol extraction (Invitrogen, Life Technologies Corporation, Carlsbad, CA). One µg of the isolated RNA was used for cDNA synthesis with a RT-PCR kit (Amersham Pharmacia Biotech, Piscataway, NJ), according to the manufacturer instructions. Quantitative real time RT-PCR was performed on a MX-3000P Stratagene instrument (Agilent Technologies, Inc., Santa Clara, CA) using specific TaqMan expression assays and universal PCR Master Mix (Applied Biosystems, Life Technologies Corporation). The following TaqMan expression assays were used in our study: Mm00434228_m1 (IL-1β), Mm00445259_m1 (IL-4), Mm00446190_m1 (IL-6), Mm00439616_m1 (IL-10), Mm00439619_m1 (IL-17A), Mm00443258_m1 (TNFá), Mm00801778_m1 (IFNã) and Mm00441724_m1 (TGFβ). Results (in triplicate) were normalized to *GAPDH* expression and measured in parallel in each sample. Data were expressed as mean fold change relative to control samples.

### Statistical analysis

Statistical analysis of differences between groups of mice was performed using the Mann-Whitney and Two-Way Anova test. Probability values <0.05 were considered significant.

## References

[1] Deterre P, Berthelier V, Bauvois B, Dalloul A, Schuber F (2000). CD38 in T- and B-cell functions.. Chem Immunol.

[2] Malavasi F, Deaglio S, Funaro A, Ferrero E, Horenstein AL (2008). Evolution and function of the ADP ribosyl cyclase/CD38 gene family in physiology and pathology.. Physiol Rev.

[3] Howard M, Grimaldi JC, Bazan JF, Lund FE, Santos-Argumedo L (1993). Formation and hydrolysis of cyclic ADP-ribose catalyzed by lymphocyte antigen CD38.. Science.

[4] Zocchi E, Franco L, Guida L, Benatti U, Bargellesi A (1993). A single protein immunologically identified as CD38 displays NAD-glycohydrolase, ADP-ribosyl cyclase and cyclic ADP-ribose hydrolase activities at the outer surface of human erythrocytes.. Biochem Biophys Res Commun.

[5] Lee HC, Aarhus R (1995). A derivate of NADP mobilizes calcium stores insensitive to inositol trisphosphate and cyclic ADP-ribose.. J Biol Chem.

[6] Schuber F, Lund FE (2004). Structure and enzymology of ADP-ribosyl cyclases: Conserved enzymes that produce multiple calcium mobilizing metabolites.. Curr Mol Med.

[7] Deaglio S, Mallone R, Baj G, Arnulfo A, Surico N (2000). CD38/CD31, a receptor/ligand system ruling adhesion and signaling in human leukocytes.. Chem Immunol.

[8] Frasca L, Fedele G, Deaglio S, Capuano C, Palazzo R (2006). CD38 orchestrates migration, survival, and Th1 immune response of human mature dendritic cells.. Blood.

[9] Viegas MS, do Carmo A, Silva T, Seco F, Serra V (2007). CD38 plays a role in effective containment of mycobacteria within granulomata and polarization of Th1 immune responses against Mycobacterium avium.. Microb Infect.

[10] Munoz P, Mittelbrunn M, de la Fuente H, Perez-Martinez M, García-Perez A (2008). Antigen-induced clustering of surface CD38 and recruitment of intracellular CD38 to the immunologic synapse.. Blood.

[11] Zubiaur M, Fernandez O, Ferrero E, Salmeron J, Malissen B (2002). CD38 is associated with lipid rafts and upon receptor stimulation leads to Akt/protein kinase B and Erk activation in the absence of the CD3-zeta immune receptor tyrosine-based activation motifs.. J Biol Chem.

[12] Munoz P, Navarro MD, Pavon EJ, Salmeron J, Malavasi F (2003). CD38 signaling in T cells is initiated within a subset of membrane rafts containing Lck and the CD3-zeta subunit of the T cell antigen receptor.. J Biol Chem.

[13] Zumaquero E, Munoz P, Cobo M, Lucena G, Pavón EJ (2010). Exosomes from human lymphoblastoid B cells express enzymatically active CD38 that is associated with signaling complexes containing CD81, Hsc-70 and Lyn.. Exp Cell Res.

[14] Cockayne DA, Muchamuel T, Grimaldi JC, Muller-Steffner H, Randall TD (1998). Mice deficient for the ecto-nicotinamide adenine dinucleotide glycohydrolase CD38 exhibit altered humoral immune responses.. Blood.

[15] Partida-Sanchez S, Goodrich S, Kusser K, Oppenheimer N, Randall TD (2004). Regulation of dendritic cell trafficking by the ADP-rybosil cyclase CD38: impact on the development of humoral immunity.. Immunity.

[16] Partida-Sanchez S, Cockayne DA, Monard S, Jacobson EL, Oppenheimer N (2001). Cyclic ADP-ribose production by CD38 regulates intracellular calcium release, extracellular calcium influx and chemotaxis in neutrophils and is required for bacterial clearance in vivo.. Nat Med.

[17] Chen J, Chen YG, Reifsnyder PC, Schott WH, Lee CH (2006). Targeted disruption of CD38 accelerates autoimmune diabetes in NOD/Lt mice by enhancing autoimmunity in an ADP-ribosyltransferase 2-dependent fashion.. J Immunol.

[18] Chen YG, Chen J, Osborne MA, Chapman HD, Besra GS (2006). CD38 is required for the peripheral survival of immunotolerogenic CD4^+^ invariant NK T cells in nonobese diabetic mice.. J Immunol.

[19] Seman M, Adriouch S, Scheuplein F, Krebs C, Freese D (2003). NAD-induced T cell death: ADP-ribosylation of cell surface proteins by ART-2 activates the cytolytic P2X7 purinoceptor.. Immunity.

[20] Chiba A, Kaieda S, Oki S, Yamamura T, Miyake S (2005). The involvement of V(alpha)14 natural killer T cells in the pathogenesis of arthritis in murine models.. Arthritis Rheum.

[21] Yoshiga Y, Goto D, Segawa S, Ohnishi Y, Matsumoto I (2008). Invariant NKT cells produce IL-17 through IL-23-dependent and -independent pathways with potential modulation of Th17 response in collagen-induced arthritis.. Int J Mol Med.

[22] Driver JP, Scheuplein F, Chen YG, Grier AE, Wilson SB (2010). Invariant natural killer T-cell control of type 1 diabetes: a dendritic cell genetic decision of a silver bullet or Russian roulette.. Diabetes.

[23] Choe CU, Lardong K, Gelderblom M, Ludewig P, Leypoldt F (2011). CD38 exacerbates focal cytokine production, postischemic inflammation and brain injury after focal cerebral ischemia.. PLoS One.

[24] Lund FE (2006). Signaling properties of CD38 in the mouse immune system: enzyme-dependent and -independent roles in immunity.. Mol Med.

[25] Guedes AG, Jude JA, Paulin J, Kita H, Lund FE (2008). Role of CD38 in TNF-alpha-induced airway hyperresponsiveness.. Am J Physiol Lung Cell Mol Physiol.

[26] Grammer AC, Slota R, Fischer R, Gur H, Girschick H (2003). Abnormal germinal center reactions in systemic lupus erythematosus demonstrated by blockade of CD154-CD40 interactions.. J Clin Invest.

[27] Cho YG, Cho ML, Min SY, Kim HY (2007). Type II collagen autoimmunity in a mouse model of human rheumatoid arthritis.. Autoimmun Rev.

[28] Kelchtermans H, De Klerck B, Mitera T, Van Balen M, Bullens D (2005). Defective CD4+CD25+ regulatory T cell functioning in collagen-induced arthritis: an important factor in pathogenesis, counter-regulated by endogenous IFN-gamma.. Arthritis Res Ther.

[29] Frey O, Petrow PK, Gajda M, Siegmund K, Huehn J (2005). The role of regulatory T cells in antigen-induced arthritis: aggravation of arthritis after depletion and amelioration after transfer of CD4+CD25+ T cells.. Arthritis Res Ther.

[30] González J, Tamayo E, Santiuste I, Marquina R, Buelta L (2007). CD4^+^CD25^+^ T cell-dependent inhibition of autoimmunity in transgenic mice overexpressing human Bcl-2 in T lymphocytes.. J Immunol.

[31] Yanaba K, Hamaguchi Y, Venturi GM, Steeber DA, St Clair EW (2007). B cell depletion delays collagen-induced arthritis in mice: arthritis induction requires synergy between humoral and cell-mediated immunity.. J Immunol.

[32] Svensson L, Jirholt J, Holmdahl R, Jansson L (1998). B cell-deficient mice do not develop type II collagen-induced arthritis (CIA).. Clin Exp Immunol.

[33] Fossati-Jimack L, Ioan-Facsinay A, Reininger L, Chicheportiche Y, Watanabe N (2000). Markedly different pathogenicity of four immunoglobulin G isotype-switch variants of an antierythrocyte autoantibody is based on their capacity to interact in vivo with the low-affinity Fcγ receptor III.. J Exp Med.

[34] Ravetch JV, Bolland S (2001). IgG Fc receptors.. Annu Rev Immunol.

[35] Neuberger MS, Rajewsky K (1981). Activation mouse complement by monoclonal mouse antibodies.. Eur J Immunol.

[36] Lubberts E, Koenders MI, Oppers-Walgreen B, van den Bersselaar L (2004). Treatment with a neutralizing anti-murine interleukin-17 antibody after the onset of collagen-induced arthritis reduces joint inflammation, cartilage destruction, and bone erosion.. Arthritis Rheum.

[37] Sato K, Suematsu A, Okamoto K, Yamaguchi A, Morishita Y (2006). Th17 functions as an osteoclastogenic helper T cell subset that links T cell activation and bone destruction.. J Exp Med.

[38] Chu CQ, Song Z, Mayton L, Wu B, Wooley PH (2003). IFNã deficient C57BL/6 (H-2b) mice develop collagen induced arthritis with predominant usage of T cell receptor Vβ6 and Vβ8 in arthritic joints.. Ann Rheum Dis.

[39] Guedez YB, Whittington KB, Clayton JL, Joosten LA, van de Loo FA (2001). Genetic ablation of interferon-ã up-regulates interleukin-1β expression and enables the elicitation of collagen-induced arthritis in a nonsusceptible mouse strain.. Arthritis Rheum.

[40] Manoury-Schwartz B, Chiocchia G, Bessis N, Abehsira-Amar O, Batteux F (1997). High susceptibility to collagen-induced arthritis in mice lacking IFN-ã receptors.. J Immunol.

[41] Vermeire K, Heremans H, van de Putte M, Huang S, Billiau A (1997). Accelerated collagen-induced arthritis in IFN-ã receptor-deficient mice.. J Immunol.

[42] Postigo J, Genre F, Iglesias M, Fernández-Rey M, Buelta L (2011). Exacerbation of collagen type II-induced arthritis in ApoE deficient mice in association with the expansion of Th1 and Th17 cells.. Arthritis Rheum.

[43] Lamacchia C, Palmer G, Seemayer C, Talabot-Ayer D, Gabay C (2010). Enhanced Th1 and Th17 responses and arthritis severity in mice with a deficiency of myeloid cell-specific interleukin-1 receptor antagonist.. Arthritis Rheum.

[44] Griffiths MM, Cremer MA, Harper DS, McCall S, Cannon GW (1992). Immunogenetics of collagen-induced arthritis in rats: both MHC and non-MHC gene products determine the epitope specificity of immune response to bovine and chick type II collagens.. J Immunol.

[45] Harrell MI, Iritani BM, Ruddell A (2008). Lymph node mapping in the mouse.. J Immunol Methods.

